# Family caregivers’ itineraries of preschool children who survived leukemia: implications for primary healthcare nursing

**DOI:** 10.1590/0034-7167-2022-0193

**Published:** 2023-02-06

**Authors:** Camille Xavier de Mattos, Liliane Faria da Silva, Tátilla Rangel Lobo Braga, Renata de Moura Bubadué, Adriana Nunes Moraes Partelli, Ivone Evangelista Cabral

**Affiliations:** I Universidade Federal do Rio de Janeiro. Rio de Janeiro, Rio de Janeiro, Brazil; II Universidade Federal Fluminense. Niterói, Rio de Janeiro, Brazil

**Keywords:** Caregivers, Child, Preschool, Leukemia, Primary Care Nursing, Qualitative Research, Cuidadores, Preescolar, Leucemia, Enfermería de Atención Primaria, Investigación Cualitativa, Cuidadores, Pré-Escolar, Leucemia, Enfermagem de Atenção Primária, Pesquisa Qualitativa

## Abstract

**Objectives:**

to analyze the path taken by family caregivers of preschool children who survived leukemia and discuss the implications for primary healthcare nursing.

**Methods:**

the narrative interview guided by a talking map and body knowledge was used with family members of children who survived leukemia, living in Rio de Janeiro (capital) and São Paulo (countryside). Conversation analysis was applied to the data.

**Results:**

five family groups (seven people) of five children started their journey in the professional subsystem of private services; four were assisted in the private sector since the onset of the illness; one was assisted in public and private services. Living conditions reduced barriers to accessing supplementary health, facilitating coordination, and listening to a reference professional.

**Final Considerations:**

the itinerary was marked by attentive listening to family caregivers by reference professionals, favoring early diagnosis, initiation of treatment, and resolution of leukemia with the cure of children.

## RESUMO

**Objetivos:**

analisar o itinerário percorrido por familiares cuidadores de crianças pré-escolares sobreviventes da leucemia e discutir as implicações para enfermagem da atenção primária.

**Métodos:**

empregou-se a entrevista narrativa orientada pelo mapa falante e corpo saber com familiares de crianças sobreviventes de leucemia, residentes no Rio de Janeiro (capital) e São Paulo (interior). Aplicou-se a análise de conversação aos dados.

**Resultados:**

cinco núcleos familiares (sete pessoas) de cinco crianças iniciaram seu itinerário no subsistema profissional dos serviços privados; quatro foram assistidas no setor privado desde o início do adoecimento; uma foi assistida em serviços público e privado. As condições de vida reduziram barreiras de acesso à saúde suplementar, facilitando a coordenação e a escuta de um profissional de referência.

**Considerações Finais:**

o itinerário foi marcado por escuta atenta de familiares cuidadores pelo profissional de referência, favorecendo o diagnóstico precoce, início do tratamento e resolutividade da leucemia com a cura da criança.

## RESUMEN

**Objetivos:**

analizar el camino recorrido por los cuidadores familiares de preescolares que sobrevivieron a la leucemia y discutir las implicaciones para la enfermería en la atención primaria.

**Métodos:**

se utilizó una entrevista narrativa guiada por el mapa del habla y el cuerpo de conocimiento con familiares de niños que sobrevivieron a la leucemia, residentes en Río de Janeiro (capital) y São Paulo (interior). Se aplicó el análisis de conversación a los datos.

**Resultados:**

cinco familias (siete personas) de cinco hijos iniciaron su andadura en el subsistema profesional de servicios privados; cuatro fueron atendidos en el sector privado desde el inicio de la enfermedad; uno fue asistido en servicios públicos y privados. Las condiciones de vida redujeron las barreras para acceder a la salud complementaria, facilitando la coordinación y la escucha de un profesional de referencia.

**Consideraciones Finales:**

el itinerario estuvo marcado por la escucha atenta de los familiares cuidadores por parte del profesional de referencia, favoreciendo el diagnóstico precoz, inicio del tratamiento y resolución de la leucemia con la curación del niño.

## INTRODUCTION

Globally, the survival rate from childhood cancer (regardless of type) could reach approximately 60% by 2030. With early diagnosis and treatment, it is possible to save more than one million lives^(1,2,3,4,5)^. Worldwide, when the diagnosis is early, treatment regimens for acute lymphocytic leukemia (ALL) in childhood can achieve a survival rate of up to 90%^(2,3,4)^. In Brazil, for the 2020-2022 biennium, it is estimated that 8,640 new cases of cancer will be registered, with 28% of the total cases corresponding to leukemia, the most common type in early childhood^(5-6)^. Early diagnosis allows the implementation of treatment regimens for ALL in childhood, with survival rates of up to 90%^(2,3,4,5)^. The speed or delay in defining childhood cancer diagnosis is directly related to the health system’s response capacity and the therapeutic itinerary followed by family members^(6)^.

The path taken by a person seeking health care is culturally influenced by their perception of health, illness and healthcare^(7)^. In turn, there is a system of cultural beliefs that contributes to people’s explanation of disease and illness in three structural domains: socio-familial (includes the social and community network), popular (lay caregivers), and professional (health team) ^(6,7,8)^. The latter corresponds to the professional subsystem and includes health professionals and the official health network^(7)^. In it, the explanations for the phenomena of illness are activated based on the manifestations presented by patients, associating with the scientific knowledge of the health professional domain. In the family subsystem, knowledge is based on experience and observation of what is repeated, what is read, seen, and heard in interaction and encounters with others (family members, friends, acquaintances, and professionals). Popular interpretations and coping strategies are transmitted from generation to generation and used by family members as explanatory models^(7)^.

The initial signs (fever, malaise, easy tiredness, and joint pain, among others) of illness caused by leukemia are similar and often confused with those of the most common seasonal viral conditions in childhood. At home, parents live with these manifestations and find it difficult to narrate them when they seek care from a health professional, which may delay the initiation of cancer investigation as part of the therapeutic itinerary. The little listening of these professionals in health services can hide more severe conditions that delay diagnosis, due to interpretations of viral conditions and or conditions common to childhood, without associating them with childhood cancer^(9-10)^.

In Brazil, the Unified Health System (SUS - *Sistema Único de Saúde*) is hybrid, and the private sector has been regulated and supervised by the National Regulatory Agency for Private Health Insurance and Plans (ANS - *Agência Nacional de Saúde Suplementar*) since 2000. Brazilian legislation (Law 12,732/2012) regulates that patients’ first treatment must be started within a maximum of 60 days after cancer diagnosis and according to the therapeutic need^(11)^. Despite this regulation, sometimes, in the itinerary of family members of children who traveled through the public sector of SUS, there are reports of unpreparedness of health professionals to suspect and refer to the specialized service network to define the cancer diagnosis. Children were misdiagnosed and treated and submitted to different procedures, in addition to the numerous trips to and from different services, which prolonged the time of disease diagnosis and postponed the start of treatment in the public network^(9,12-13)^.

In Kenya, family members whose children were cared for in a publicly funded hospital complained about long delays in care, fear of treatment and its side effects, and fear of surgery^(14)^. Most family members of children with cancer did not have health insurance before the disease was diagnosed, nor were they treated for the first time at the reference hospital. They bore high hospital and travel costs, exposing themselves to precarious transport between the homes farthest from the hospital. The few families that had health insurance were diagnosed and treated more quickly^(14)^. In Jordan, among the needs ranked by family members of children treated at a tertiary oncology hospital, trust in the health system stands out^(15)^.

However, little is known about the therapeutic itinerary of family members of preschool children who survived leukemia, from the diagnostic investigation stage, in the private sector of SUS. More studies are addressing the route taken by family members in the public sector in investigating suspected cases and leukemia diagnoses^(9,12-13,16)^ than in the private health sector^(10,14)^.

## OBJECTIVES

To analyze the journey taken by family caregivers of preschool children who survived leukemia and discuss the implications for primary care nursing.

## METHODS

The writing of this article was guided by the Consolidated criteria for reporting qualitative research (COREQ) checklist for qualitative studies.

The results section of the article was extracted from the database of a nursing dissertation “*Necessidades de saúde de familiares com leucemia: conversas e metáforas no itinerário de cuidados*”, authored by the first author, under the supervision of last author, in the Graduate Program in Nursing, *Escola de Enfermagem Anna Nery, Universidade do Rio de Janeiro* , 2017. The data analyzed in this article could be retrieved from the *Universidade Federal do Rio de Janeiro* Minerva Database of Theses and Dissertations: http://objdig.ufrj.br/51/teses/858934.pdf.

### Ethical aspects

The Research Ethics Committee approved the research of the proposed institution. After reading, doubts regarding the research and the Informed Consent Form were clarified. Before starting the interview, all participants signed the Consent Informed in a reserved and private place. To anonymize personal information, the following identification was adopted in the excerpts: M1. Mother and stepfather; M2. Mom and dad; M3. Dad; M4. Mother; M5. Mother.

### Study design and methodological procedures

The narrative method of qualitative research was chosen, aiming that a person was the narrator of their own experience in producing the text to be interpreted. The narrative is critical for nursing research, as it reveals and mobilizes the possibility of solving problems emerging from qualitative evidence. Narrators interpret their historical and sociocultural potentialities and limitations, taking their biography as a reference point^(17-18)^.

The in-depth interview technique was implemented in five phases: preparation for the interview; interview topic initiation; central narration with free speech about the topic; time for questions to add something unspoken; interview conclusion (interruption of recording, thanks and registration in field diary).

The creativity and sensitivity techniques (CST) talking map and body knowledge were adopted to conduct in-depth interviews. As arts-based research strategies, they favor access to human subjectivity with illness experiences and the construction of narratives ethically in approaching sensitive topics^(19)^. The body map and the speak map are metaphors that produce meanings in narrative communication, cornerstones in the narration of signs of illness in the body, places, and people searching for health care. At the end of each interview, the interviewer summarized the narrated topics so the interviewee could validate them. These topics represented the starting point for empirical material analysis.

In applying both techniques, their purpose was explained, providing materials (sheet of A4 paper, colored pencils, crayons, and colored pens). The triggering question was presented: for the map - please, draw the places and people who participated in your pathway, from the first signs of illness of (...); for the body: from these keywords, select and locate in the body those that express what most caught your attention in the course of (...) illness. In the third, the interviewee elaborated on the artistic production; at the last moment, he was asked to speak freely about what he had done.

As childhood cancer is a sensitive topic, addressing latent memories of this illness itinerary requires a solidary attitude. In the initiation phase of the in-depth narrative interview, CST talking map or body of knowledge was applied^(19)^. However, when two participants belonged to the same family, the talking map was applied to the first interviewee and the knowledge body with the second, designated by him. When the interview was carried out with only one person, only a speak map was applied to initiate the interview.

Each interview lasted between 60 and 110 minutes, was recorded on a digital device, carried out between June and September 2016, and transcribed verbatim for a study completed in 2017.

### Study setting

Fieldwork took place in a place chosen by participants, which could be at the interviewee’s residence or workplace in Rio de Janeiro city. The researchers (first and last author) conducted the online interview by videoconference with a participant who lived in a municipality in the state of São Paulo.

### Data source

The snowball was applied as a technique to access potential research participants^(20)^. The first participants indicate, among their acquaintances, new volunteers with the potential to participate in the research because they share the same experiences related to the investigated phenomenon. In sampling, the researcher asks the first respondents if they know someone with similar opinions or situations to participate in the research.

To operationalize the technique of snowball, participant recruitment took place in virtual communities and blogs of family members of children with leukemia. On those social networks, they recorded their stories as mothers and fathers of children diagnosed with ALL in the first five years of life. For those who provided e-mail on those networks, an e-mail was sent inviting them to volunteer for the research. At the same time, they were asked to indicate people they had close relationships with. We included: a) family caregivers of children diagnosed with ALL in the first five years of life; b) people who shared the itinerary of seeking healthcare, over 18 years of age; c) good communication skills (verbal preserved to narrate), and motor coordination (to writing and drawing). The potential participant must also freely navigate the internet and skills with digital inclusion tools (blog, social networks, e-mail, etc.)

We excluded family caregivers of children with genetic syndromes (Down Syndrome) who became ill due to ALL, family members of children in palliative care, and accompanying hospitalized children.

Of the 13 people able to participate in the survey, seven belonged to five families with five children who followed the itinerary in supplementary health. Six were gathered on a social network and one by a snowball.

### Criteria for closing field work

Theoretical saturation was adopted as a criterion for closing the fieldwork - when no new element is added to what has already been narrated by other participants^(21)^. Thus, the same pattern of narratives was observed in the sixth person interviewed (from three family groups), deciding to maintain the seventh interview.

### Data analysis

The data were submitted to the conversation analysis technique, seeking to sequentially organize the turns of speech based on understanding the meaning of the first turn concerning the subsequent turn^(22)^. Following these recommendations, the procedures were operationalized in five stages: intelligibility of narrated text, sequence of events, chronological ordering of conversation turns, axial coding, indexing of themes, and reduction. The narrated text was first adapted to the syntactic language for fluid reading. In the second, the narrated text was sequentially reorganized to highlight the temporality attribute of the events and happenings to show the narrator’s interaction with the events marked in the life story. In the third, the time of each event was considered to order in conversation turns, based on initiation, presentation of the narrated topic, conflict, and outcome. In the coding movement, similar and different conversation turns were grouped by interviewed individuals (in the same nucleus of children’s family members). The common meanings from different family groups were regrouped and indexed by similar conversation units to constitute themes. The conversation turns’ text was reduced to the central core of meanings representative of the interviewee’s narrative set, resulting in an axial codification with 37 turns of conversation, reorganized into themes, understood in the light of the therapeutic itinerary theoretical framework^(7)^.

## RESULTS

Five family nuclei had five children, composed of seven people. They belong to families with mixed (mother and stepfather) and nuclear (mother and father) structures. Three participants were male (two fathers and a stepfather), and four were female (mothers); all had completed higher education (physician, engineer, business administrator, economist, lawyers, and real estate broker); and had high-paying jobs or were micro-entrepreneurs. Four family nuclei belonged to the upper social class, with an income above 20 national minimum wages (NMW) (1 reference NMW in the year of implementation of this research fieldwork = R$880.00 (about US$160.00), residing in upscale neighborhoods in the south zone of the city of Rio de Janeiro, whose square meter was equivalent to R$21,608.00 (about US$3,928.72).

The fifth family nucleus belonged to the middle class, with an income of up to 10 NMW, and lived in an industrial city in the countryside of São Paulo. All had health insurance, and one temporarily accessed the SUS public network to treat their child. ALL diagnosis was defined in a girl, infant (1 year and ten months), three girls, and one boy of preschool age (between two years and nine months and five years and seven months of age). Three children were diagnosed between days 10 and 20 of the onset of the first manifestations (girl 4, on day 10; girl 2 and boy 5, on day 15), and two, over 20 days (girl 1, on day 27; and girl 3, on day 21).

### An explanatory model of the family subsystem in the therapeutic itinerary of children with leukemia

Family caregivers’ narratives triggered explanations about the onset of the itinerary and signs of illness in the child, involving family caregivers’ perception of the itinerary onset. Child behavior change, fever, purple spots, and constant body aches were graphically represented in a talking map and knowledge body, mobilizing the initiation of their narratives. When describing the scene produced in talking map and body knowledge, they presented characters from their stories restricted to their family’s (child, mother, father, stepfather) and collaborators’ (nanny) primary nucleus. The house, the restaurant, the carnival ball, the circus, and the vacation trips placed their narratives spatially in time. In the family system, temporality indicates an illness onset in periods of barrier to personal access to the professional system due to special celebration dates (Christmas, New Year, school and family vacations, and Carnival).

*In the week of Christmas* [2009] *at home, she* [/] *with a low and continuous fever, which she gave in with antipyretic and analgesic, weakened, constant pain in her leg* [...]*. For a week, she was prostrate, sad! Everything was programmed for two trips. On the 27th* [December], *I went to a beach in Brazil, and it was seven days of sun, fun, and friends. On New Year’s Eve, she got asleep more than usual and slept on her nanny’s lap. Two days later, we returned to Rio, got ready, and went skiing for the first time! Again, she was more tired than usual.* [meanwhile, the family took the girl to the appointment] *The pediatrician said, “It must be the usual, the throat! Give me medicine”. She took it and got better.* (M1. Mother and stepfather)

At the onset of the illness, fever and pain episodes were controlled by using antipyretics and analgesics during the Christmas holidays and New Year’s Eve, which postponed the diagnostic investigation for two weeks.

*In December 2014, he started with a fever of 38ºC* [...] *I gave him the antipyretic; he got well, and then the fever came back* [...]*. He was sick for two weeks; he had nausea, anemia* [pallor], *a lump on his neck behind his ear, and very quiet and pouting.* (M5. Mother)

Another family nucleus narrated changes in a behavior pattern, including cyclic fever and abdominal distension, leading them to contact a reference health professional (pediatrician) by telephone in search of a solution to their child’s health problem. Although, on vacation, she had left a system of care.

[...] *on a Sunday in January* [2008], *she* [aged one year and ten months] *had prostration and the first week-long fever cycle - two days with 37.3ºC and 37.5ºC - constant, which subsided with antipyretic, two to four days without fever, with improvement in prostration. She played, went to bed with much fatigue and lack of energy, and slept a lot* [...] *she woke up at 11 am, noon! The distended belly. In the other two-day cycle, again the fever, she slept because she was prostrate. She took antipyretics and got better; we were adapting. My wife said, “It’s not cool!”. It was on January 21st [2008] when I noticed her belly was more swollen!* (M2. Mother and father)

In another narrative, changes are observed in a pattern of emotional behavior, a manifestation of regressive behavior and greater affective dependence with fever and cries of pain when walking, something unusual for a child who was already walking without help. These situations were perceived at home, at a restaurant, and at a children’s carnival ball. This happened during a long weekend, making it difficult to access professional in-office care in the private health system.

*It all started on Thursday, February 11th* [2010], *with fever without cause; she was unwell and slept cuddled with me in bed. The next day, she became discouraged, despondent, and needy and began to feel pain in her leg. On Sunday* [14], *she just wanted to stay in the stroller. On the 20th* [Saturday of Carnival], *he woke up but did not get out of bed; he was screaming in pain when walking! I said, “Dad, call the pediatrician and tell him he has to see you today!”. He doesn’t see the office on Saturday!* (M4. Mother)

A girl’s illness onset (3 years and one month old) was marked by objective signs of high and continuous fever, a decline in her general condition, and changes in behavior (asthenia and irritability). The narrative of purple spots on the legs and dorsal region by her father, who was a physician, was added to the observation of change in behavior by her mother so that family members could start the therapeutic itinerary.

[...] *she had been sick for a month* [started in May 2012], *had a fever, was asthenic, and took a long time to recover. She had a fever peak without major complications. Then she had two days of high fever and a general decline and nothing else* [...] *it passed* [...] *she was irritated but had no other symptoms. My wife noticed that she was different. When I left the house, I knew it was not a good thing, and she had bruises on her legs and back. I was pretty sure it was leukemia. I already had an idea of what it could be, and I took her to the hospital* [...]. (M3. Father)

Manifestations associated with fever, change in the child’s behavior (prostration, asthenia, irritability, excessive sleepiness), pain and bruises led family members to seek care with physicians from their support network: in the supplementary health system of their health insurance network. Therefore, a beginning of itinerary in the professional subsystem.

### An explanatory model of the professional subsystem in the therapeutic itinerary of children with leukemia

Welcoming the sick child by a reference health professional (a pediatrician or a substitute) from the private sector corresponds to the first response to solve the problem in the first access to the professional subsystem. Fever was a symptom rationally interpreted as a manifestation of a viral infection, a common illness among children in infancy (under two years of age). For these conditions, treatment was symptomatic (with antipyretics), and the family watched the child closely.

[...] *January 20^th^, the pediatrician was traveling and left an assistant we didn’t know. He examined her and said, “Look, it must be a virus* [...] *see if the fever will decrease* [with antipyretics] *or not, or if it will improve!”.* (M2. Mother)

The same pattern of the narrative of another girl’s family member (3) is observed about what was explained to them in the professional subsystem care itinerary for high and nonspecific fever associated with the virus.

*The pediatrician saw the girl 15 days before diagnosis and said it was a viral infection of high and unspecific fever.* (M3. Father)

Since an explanation based on their subjectivity, difficulty in walking, and constant leg pain escapes the family members, they seek the professional subsystem with explanatory models specific to this system and outcomes in the resolution that corresponds to them.

[...] *over the phone, I told the pediatrician that her pain was not getting better, and I said, “Dr., this is not normal!”. He responds, “* [...] *must have sprained.” and asks, “Is she walking?”* [...] *she limped and walked and limped. I said: “No, she must have cracked* [fractured] *something”. He reassured me by saying, “No, it must be nothing* [...] *it will pass! Give me a pain reliever!”. Moreover, I kept insisting, “No, she must have been hurt* [...]*”. He replied, “So* [...] *I’m going to get an x-ray* [...] *I’m going to ask a friend from orthopedics to see her”. On Tuesday* [16] *of Carnival, he took the X-ray, and nothing!* (M4. Mother)

These manifestations were clinically investigated in medical anamnesis to prescribe therapies, request and refer for exams, even in a period of difficult access to health services in private health network clinics. In this regard, pain symptoms when walking and limping gait are triggers for formulating diagnostic hypotheses to be tested with scientifically based exams and opinions. The meeting of family members’ narratives with those of the professional, mediated by attentive listening and available on the telephone and face-to-face channels, accelerates the conclusion that it is not a growing pain, indicating that the investigation must continue.

*The orthopedist said, “It must be a growing pain because it hurts more at night; let’s see!”. Indeed, at night she screamed more. Sometimes the pain would get worse during the day, then she would lie down, and the pain would go away. She returned to the orthopedist on Thursday with my father* [maternal grandfather]*. On Friday, when I got home from work, she came running without a limp and hugged me* [...] *how wonderful! She said, “Mom, I’m great. It’s all gone!” and on Saturday* [20], *she woke up without walking.* (M4. Mother)

Searching for a care itinerary continues whenever the disease condition does not have a resolute outcome. The private health system shortened the time of care, whose communication channel was mediated by the referral pediatrician’s availability.

In another set of family narratives, physical assessment in a face-to-face consultation allowed identifying an enlarged organ, especially splenomegaly, with recurrent cycles of fever associated with the need for diagnostic investigation.

*I called the pediatrician, who had returned from a trip* [the 20^th^], *to schedule an appointment. I left work* [the 21^st^] *and took my daughter to the appointment. He felt the distended spleen. I thought, “What must it be? I had already suspected!” ... after the second fever cycle, on Monday* [21^st^], *we went back to the pediatrician, and he said, “I think the organs* [...]”. (M2. Mother and father)

From another family member’s narrative, that of a boy aged three years and ten months, episodes of persistent fever stand out as a justification for seeking emergency care in two moments; however, without the expected resolution.

*In the first week, we went to the emergency room. The physician gave me an antipyretic* [...] *another medicine to take for seven days. Nevertheless, he continued with the same symptoms, fever* [...]*. I came back and had the misfortune of being seen by the same physician on the other shift. The physician said, “It’s no big deal! It’s a lump!”* [...] *and she prescribed an antibiotic* [...] *I told my husband, “I’m not going to buy it because that’s not right”.* (M5. Mother)

The transition from family to the professional system is narrated as the beginning of the care itinerary. For a group of family members, caring for a child was not individually personalized. They felt they needed to be more confident in the competence of the health professional who assisted her due to a lack of attentive and qualified listening.

Families sought a pediatrician, by phone and in-office consultation, integrating primary healthcare of the Brazilian supplementary health system to understand what was happening to their children, prescribe medication, and request laboratory and radiographic exams.

*On Sunday* [January 17, 2010], *I called the pediatrician, explained the clinical picture, and he said, “Do a blood test early tomorrow; I’m apprehensive about dengue!* [...] *I want a complete blood count!”. The exam was performed in a private laboratory next to my house. By the end of the day, he called me to let me know his platelets were at 17,000/150,000. In the evening of the same day, the pediatrician came to my house and asked for a new blood test. The clinical examination did not match her condition or the test result. On the 19th early, a new exam was performed. Her platelet was at 11,000 overnight.* (M1. Mother and stepfather)

The laboratory network, contracted by health insurance, provides timely test results in the professional subsystem. The objective parameters of blood count show thrombocytopenia that rules out the first suspicion of dengue. The relationship between the child’s parents and the pediatrician was favored, as he has monitored her since birth. In the face-to-face consultation, new objective pathophysiology parameters (sternum, liver, and spleen palpation) were incorporated into the explanation for the need to broaden the investigation about what was happening to the child with constant pain in the legs and difficulties in walking.

[...] *she would have had an enlarged spleen and liver and pain in her sternum and legs. Pains in her legs were the only ones she constantly had* [...] *she didn’t have pain in her diaphragm* (chest). *He was squeezing her sternum, a sign that she might have the disease, and she didn’t respond, palpating her spleen and liver; they were normal sizes!* (M1. Mother e stepfather)

The children referred to the supplementary health referral hospital underwent clinical, laboratory, and laboratory tests (leukometry, spinal puncture) and were assessed by specialists. The objective parameters (blood count results, leukocyte count of 70 thousand, myelogram, immunophenotyping) was decisive for the diagnostic definition.

*He asks to go to the hospital. I was hospitalized on a holiday eve* [January 20^th^]. *The hematologist did the clinical examination that it hurt* [...] *and scheduled the spinal puncture for the following day.* (M1. Mother and stepfather)

*On the second visit to the pediatrician, he found the liver and spleen distended and said. “I’m finding it weird; if it’s a virus. I’d like you to do a blood test. It could be a solid viral* [frame]. *It could be mononucleosis! Let’s take a deeper look tomorrow”. When she did leukometry, she had an absurd count. He sent it to the hospital, called a hematologist to do a myelogram and a spinal puncture, and there was a chance it was leukemia.* (M2. Mother and father)

Deepening the investigation contributed to a quick diagnostic definition and treatment initiation under the child’s pediatrician coordination.

*The diagnostic phase was very fast* [...] *5 to 7 days, from the first symptom to starting treatment. We think this helps a lot in the prognosis of cure.* (M2. Mother)

The professional training of the child’s father as a physician and his network of professional contacts mobilized the investigation of the first clinical manifestations (purple spots on the body), which called the parents’ attention to seeking care with the child’s pediatrician.

*In June 2012, she started with purple spots on her legs and back. Her pediatrician was sick and said, “Take her to the hospital; I’ll call there”. They had two pediatrician friends who worked there. We took the blood test and went home. My friend called me and said, “Come back here; the exam didn’t go well!”. She asked for further tests. Our saga has begun. We were hospitalized for about 10 days. Take a test, a spinal puncture, that whole protocol* [...]. (M3. Father)

As for the family member of girl four, the itinerary in the professional system began with suspicion of leukemia due to intense pain in her leg and difficulty standing up and walking. Telephone contact with the pediatrician marked the beginning of the itinerary in the professional subsystem.

*I said, “Dad, ask him if leukemia causes leg pain”. He* [the pediatrician] *answered the father’s question, “It can, but a thousand things can cause it!”. At the clinic, he examined, “I have no idea. I can only say that it is dire! It’s not a growing pain; I need to check it out!”. He said, “No child screams like that! The pain she has is severe; it could be rheumatoid arthritis”, then she asked for laboratory and state tests, “You will do it on Monday; ask for home collection”. I asked, “What do I do with her?”. He said, “Give her a painkiller!”. I said, “Isn’t she going to take an antibiotic? A corticosteroid, an anti-inflammatory? Will she be in pain? She does not walk!”* [...] *I left there devastated! On Monday morning, he made a home collection for the blood test for the first time. Dr. X called me when we were walking around the church at night. He said, “I made an appointment with a hematologist for tomorrow* [...] *I’ll send her the test results”.* (M4. Mother)

*My son was no longer able to walk. The pediatrician assessed and asked for blood, feces, and urine tests; she received the results on her cell phone and called my husband. The blood test was altered, and it was to be repeated. The next day, we had the second blood test. In the afternoon, she received the result again by cell phone and said that she needed to talk to us and that it was to go to the hospital.* (M5. Mother)

As a care coordinator, the reference professional dialogues with the child’s family caregiver communicates the test result and forward it to specialized care. In this way, it prioritizes the child’s health needs, reduces time and barriers to access, to proceed with the diagnostic investigation with a specialist from its network of professional relationships. In turn, the speed of the initial care (complete anamnesis, physical examination, and interpretation of laboratory test) coordinated by the reference professional (the pediatrician) shortened the consultation time with a specialist, a perspective of essential longitudinality for early childhood cancer diagnosis. The following steps - requesting and scheduling the myelogram - were decisive for the outcome of the leukemia diagnostic investigation itinerary. The family member of the only boy, like the other children whose family members used the supplementary health system, had a reference professional as the care coordinator to request tests, provide results over the phone and ask for hospitalization.

## DISCUSSION

Family members of children with ALL belonged to the upper class, with socioeconomic conditions and maintenance of a standard of living that allowed them to access the supplementary health system through health insurance with broad coverage. All had a high level of education and lived in neighborhoods with easy access to the supplementary health service network (pediatricians’ offices, laboratories, specialized hospitals). These aspects facilitated starting the therapeutic itinerary and defining the disease diagnosis in early childhood between 10 and 27 days after the onset of the children’s illness. These material conditions of existence guided sequential and chronological narrative constructions about the illness of these infants and preschool children.

Almost a quarter of the Brazilian population (48 million people) has access to private health plans and insurance whose assistance coverage is offered by operators through individual and collective plans. There is also a wide range of private services, not limited to health plans and insurance, individually covered by private contractors. The SUS public network also contracts private and philanthropic services in a complementary way. Primary care is the municipality’s reference unit for articulating and referring people who need specialized care. It exists in the public and private sectors of supplementary health. However, due to the high demand for care, there is a long wait for consultations or specialized tests, leading family members to seek this type of care in private health plans^(9,12-13)^.

In this way, explanatory models about illness and decision-making to proceed with the therapeutic itinerary, from the family subsystem to professionals, acquire personal and social meanings in the disease experience for different family groups. The beginning of the diagnostic investigation itinerary was postponed due to the private health sector’s limited individual consultation care provision. Another issue for delaying the diagnosis was during long holidays (Christmas, New Year, summer holidays, and Carnival). Children whose manifestations of the first signs and symptoms of illness occurred during those times had difficulty accessing doctor consultations.

Recognizing that family members are the ones who best know children’s habits and behaviors helps health professionals in search of a resolution for what is happening to the child. These people can notice any changes in children’s health status^(9,16)^.

Thus, the narrative of temporality indicates an onset of illness with a partial barrier to accessing the professional system, as the pediatric physician did not attend on long weekends. The access of children’s family members to pediatricians, the network of laboratory services, and specialized and hospital care shortened the diagnostic investigation time for those children who fell ill in periods of regular calendars.

The family members’ perceptions materialize in the initiation of the narrated central theme, a sensitivity initially identified by the narrator’s look at the change in children’s behavior and the presentation of signs and symptoms. Temporarily, the family belief subsystem was supported by professionals. So, family members maintained their life projects (family vacation trip, business trip, trip to the circus) and postponed the beginning of the diagnostic investigation of episodes of persistent fever, drowsiness, fatigue, and bone pain. However, this onset was not late and allowed the diagnostic definition to occur in the first month of the children’s illness.

In the public sector of SUS, access to leukemia diagnosis and treatment itinerary is sometimes challenging due to the high pressure of demand. However, in the private supplementary health sector of SUS, there is a shortening of the service time whose communication channel was mediated by the availability of a reference professional (pediatricians). In the pediatric consultation via call center or face-to-face emergency, identifying clinical evidence (X-ray), with the physical examination propaedeutics, were organized and interpreted to suspect common conditions (sprain, growing pains, virus), dengue, toxoplasmosis, and rheumatic fever.

Even though childhood cancer is the second leading cause of mortality among children under five years of age, it was the last frontier of suspicion to initiate a diagnostic investigation^(23-24)^.

The link between the reference health professional, the child, and the family member helped coordinate this care, with outcomes for faster referral to specialized care, an abbreviation of the time between requesting tests and contact with the specialty for hematological disease diagnosis. The results of this study point to the absence of barriers to accessing diagnostic investigation of childhood cancer.

The narratives reveal the longitudinality and coordination of care, in the SUS supplementary health, by the pediatrician available for telephone service and in the pediatric consultation, with access to laboratories for carrying out tests. In contrast, in Brazil, the path taken by family members in health services, whether public, at different levels (primary, secondary and tertiary) and SUS care networks (primary, urgency, emergency, medium, and high complexity) reveal barriers of access to early cancer research. Often, these caregivers live an exhausting pilgrimage in search of care^(6,9)^.

Coordination of care means communicating between the different points of care and taking responsibility for the care of users through a horizontal, continuous, and integrated relationship to produce shared management of comprehensive care. Longitudinality of care presupposes continuity of care through bonds, and effective clinical interventions centered on the person, to increase the degree of individuals’ autonomy and social groups in the health system^(25)^.

In the therapeutic itinerary professional subsystem, the explanation of disease is based on five parameters - cause (etiology), symptoms (manifestation and time of onset), pathophysiology (systems and organs affected), evolution, and treatment. Relevant clinical evidence is organized and interpreted through scientific rationality, determining among the available therapies the best and safest for patients and clinicians^(9)^.

In the supplementary health system, the suspicion of the unusual by listening to anamnesis and clinical examination and requesting complementary tests (laboratory and imaging) help define a cancer diagnosis. The language of rationality used by health professionals contributes to defining the diagnosis and explains children’s treatment. With the current network of services available, the first physician to be sought out by children’s families is a generalist pediatrician or general practitioner physician who regularly works with the Family Health Strategy (FHS) of the Primary Healthcare network. Whether in the public or private sector of SUS, pediatricians are responsible for identifying suspected cases, initiating additional investigations, and referring children or adolescents to the reference service^(16)^.

In Kenya, after noticing the symptoms of children with cancer, 58 parents (59%) sought alternative treatment for their children due to the low cost, hope for improvement and cure, the recommendation of their support network, easy access and child maintenance in the family. However, the use of alternative medicine resulted in a longer delay in defining the diagnosis^(14)^. The precocity and speed with which these procedures must proceed are decisive for children with leukemia to receive successful treatment and achieve the best cure rates.

### Study limitations

There are geographical limits, as participants lived in two cities in two states in southeastern Brazil, which are economically more industrialized, with a higher concentration of consumers of private health plans with a more significant offer of private services. Methodological limits were not listening to the narratives of other family members of the same children or health professionals who assisted them. The racial limit refers to the fact that none of the interviewees or children belonged to the black/brown race/color.

### Contributions to nursing and health

Listening to family members’ narratives can be a working tool for nurses in primary healthcare services, whether in the private or public health sector. Therefore, one needs to be attentive to family members’ perceptions about the first signs of illness in children with leukemia and not just listen to reports of complaints. A professional who is the care coordinator can guarantee comprehensive care, recognize the severity of children’s clinical manifestations, carry out a complete anamnesis, suspect diseases, request laboratory tests, etc. Continuing to listen in consultations with shorter intervals can help differentiate the manifestations of the most common diseases in childhood from those that may lead to suspicion of childhood cancer. Therefore, attentive and sensitive listening to the narratives of family members of children with leukemia helps nurses and other health professionals to suspect cancer and initiate the diagnostic investigation as part of the therapeutic itinerary. The need to value the illness process narrative is highlighted as an essential element to expand therapy in nurses’ clinical practice based on comprehensive health care for children.

## FINAL CONSIDERATIONS

The itinerary of family members of children with leukemia in the supplementary health system began with the first signs of illness. Consistent, regular, sequential, and temporal narratives of these caregivers were listened to carefully by the reference professional (pediatric physician). That professional maintained a bonding relationship leading to acting as a care coordinator to constitute a line of clinical reasoning that included childhood cancer suspicion in sequential listening. However, children’s growing pains (bone and muscle) and intermittent fever were initially interpreted as a symptom of childhood viral diseases or possible complex diseases, not cancer. Many childhood complex disease mentioned by family narratives were rheumatic fever, toxoplasmosis, dengue fever, and infectious disease mononucleosis.

Bonding, longitudinality, and coordination of care are attributes of primary care that favor the approach of comprehensive care and help to minimize barriers to accessing the health system, whether private (supplementary) or public. In this sense, open reception in primary care in supplementary health created a direct line from children’s families to reference professionals, offering favorable conditions for childhood leukemia diagnosis based on listening to a temporal and sequential narrative.
